# Eye Size at Birth in Prosimian Primates: Life History Correlates and Growth Patterns

**DOI:** 10.1371/journal.pone.0036097

**Published:** 2012-05-02

**Authors:** Joshua R. Cummings, Magdalena N. Muchlinski, E. Christopher Kirk, Susan J. Rehorek, Valerie B. DeLeon, Timothy D. Smith

**Affiliations:** 1 Department of Biology, Slippery Rock University, Slippery Rock, Pennsylvania, United States of America; 2 Department of Anatomy and Neurobiology, University of Kentucky, College of Medicine, Lexington, Kentucky, United States of America; 3 Department of Anthropology, University of Texas, Austin, Texas, United States of America; 4 Center for Functional Anatomy and Evolution, Johns Hopkins University School of Medicine, Baltimore, Maryland, United States of America; 5 School of Physical Therapy, Slippery Rock University, Slippery Rock, Pennsylvania, United States of America; 6 Department of Anthropology, University of Pittsburgh, Pittsburgh, Pennsylvania, United States of America; University of Montréal and Hôpital Maisonneuve-Rosemont, Canada

## Abstract

**Background:**

Primates have large eyes relative to head size, which profoundly influence the ontogenetic emergence of facial form. However, growth of the primate eye is only understood in a narrow taxonomic perspective, with information biased toward anthropoids.

**Methodology/Principal Findings:**

We measured eye and bony orbit size in perinatal prosimian primates (17 strepsirrhine taxa and *Tarsius syrichta*) to infer the extent of prenatal as compared to postnatal eye growth. In addition, multiple linear regression was used to detect relationships of relative eye and orbit diameter to life history variables. ANOVA was used to determine if eye size differed according to activity pattern. In most of the species, eye diameter at birth measures more than half of that for adults. Two exceptions include *Nycticebus* and *Tarsius*, in which more than half of eye diameter growth occurs postnatally. Ratios of neonate/adult eye and orbit diameters indicate prenatal growth of the eye is actually more rapid than that of the orbit. For example, mean neonatal transverse eye diameter is 57.5% of the adult value (excluding *Nycticebus* and *Tarsius*), compared to 50.8% for orbital diameter. If *Nycticebus* is excluded, relative gestation age has a significant positive correlation with relative eye diameter in strepsirrhines, explaining 59% of the variance in relative transverse eye diameter. No significant differences were found among species with different activity patterns.

**Conclusions/Significance:**

The primate developmental strategy of relatively long gestations is probably tied to an extended period of neural development, and this principle appears to apply to eye growth as well. Our findings indicate that growth rates of the eye and bony orbit are disassociated, with eyes growing faster prenatally, and the growth rate of the bony orbit exceeding that of the eyes after birth. Some well-documented patterns of orbital morphology in adult primates, such as the enlarged orbits of nocturnal species, mainly emerge during postnatal development.

## Introduction

Compared to other mammals, primates tend to have large eyes relative to head or body size [1]. Interspecific variation in adult eye size among primates has been attributed to differences in ecology [2], [3] and allometric factors [4]–[6]. The importance of the size of the eye to primate facial form, including orbital orientation, has been extensively discussed [5]–[7]. During fetal development the relatively large primate eye profoundly influences facial form, perhaps more so in small-bodied nocturnal species [8]. At the present time, however, growth of the primate eye is only understood in a few primate species.

There is a reasonable basis to expect eyes to grow either in a “somatic” rate (in tandem with head or body size), or in a “neural” pattern (e.g., in tandem with brain development). Embryologically, parts of the eye (e.g., the connective tissue tunics) are derived from mesoderm [9], [10], and thus eye growth might be expected to closely follow that of other somatic structures rather than visceral or neural structures. Other parts of the eye (e.g., the retina) develop as an outgrowth of the neural tube [9], [10]. As a result, eye growth might instead be predicted to mirror developmental patterns observed for the brain, such as a relatively large extent of prenatal growth [11]. Somatic and neural growth patterns of primates have received much consideration in the literature, with anthropoids being the best studied by far. Compared to other mammals, anthropoids postpone somatic growth via a slow rate of preadolescent growth [12]. Neural development is also prolonged in anthropoids [12], but most neural growth occurs prenatally – in other words, most newborn anthropoids have brains that are more than half of the adult volume [11], [13]. Compared to anthropoids, the amount of prenatal brain growth is less in strepsirrhines (lemuroids and lorisoids) [11]. Our knowledge of eye growth is meager by comparison to brain growth, and heavily biased toward anthropoids (humans and macaques in particular – [14]–[17]; but see [8] regarding fetal growth of the eyes in *Tarsius bancanus*). In particular, the growth and development of the eye and orbital region in non-anthropoid primates is very poorly understood.

Mammalian species differ in how well-developed neural, muscular, and other tissues are at birth. Primates are generally considered precocial compared to other mammals [18], [19], but neural and somatic development is not synchronized. This pattern is evident in newborn primates. Although eyes are open at birth or days later, infant primates vary in muscular development and duration of dependency [20], [21]. Thus it is reasonable to ask whether eye growth varies according to developmental parameters other than body size, such as life history or ecological characteristics. Gestation length and weaning age are important life history variables because they reflect the pace of development and duration of dependency, respectively. These life history traits relate to neural development in important ways. The variation in the relative length of gestation, for example, may allow us to clarify the timing of eye growth in primates (e.g., is eye growth occurring more prenatally or postnatally?). Weaning age appears to relate to the pace of brain growth, at least in some primates. Among anthropoids, species with brain growth that ceases early tend to have a later weaning age [22].

Here we seek to better understand primate eye development through an interspecific comparative study of the eye and orbital aperture in 18 prosimian primates (17 strepsirrhines and *Tarsius syrichta*). The first goal of this analysis is to better understand the timing of eye and orbit growth during prenatal versus postnatal ontogeny. The extent of eye and orbit growth that is achieved by birth is inferred by comparing eye and orbit diameter of neonates to adults. The second goal of this study is to investigate whether relative eye size (eye diameter, controlling for cranial length) at birth is influenced by life history or ecology. Specifically, we examine the relationship between relative eye and orbit diameter and somatic measurements, activity pattern, relative neonatal mass, gestation length, and age at weaning.

## Materials and Methods

### Sample

Forty-three primate cadavers were examined. Of these, 38 infants that died on postnatal day 0 to day 8 were used for statistical analyses of osseous and soft tissue dimensions at birth (Table 1). Four other subadult and infant specimens were examined for comparative purposes (see below). One adult specimen was also used for measurements. The adult specimen was a cadaveric head of *Otolemur garnettii* stored in formalin. The source of this sample was an animal that had been euthanized after use in studies, unrelated to the present one, at Duke University Medical Center (see [23], for details regarding this research). Infant cadavers were obtained from the Duke Lemur Center, except for one infant *Eulemur mongoz*, which was obtained from the Cleveland Metroparks Zoo. All had been previously fixed in 10% buffered formalin, or were received frozen and fixed upon delivery. Specimens were selected from a larger sample of infant cadavers currently housed in the laboratory of TDS that either died postnatally or were stillborn. There are inherent difficulties in knowing the precise somatic age of infant primates (see discussion by Smith and Leigh, 1998 [24]; Smith et al., 2011 [25]). To minimize the possibility of including underdeveloped perinatal specimens in our analysis, fetuses or stillbirths that were clearly premature were excluded from the study. Prematurity in stillbirths was assessed based, in part, on crown-rump-length compared to other specimens of the same species in this sample or the literature. In addition, lack of body fur in some species (excepting cheirogaleids, which uniformly had minimal fur covering) was used as an indicator of prematurity.

**Table 1:** Cranial length and life history variables of the specimens used in statistical analyses.

Taxonomic group	n	Activity pattern^1^	CL	Neonatal mass^2^	Gestation^2^	weaning age (days)^2^
**STREPSIRRHINI Lemuriformes Lemuridae**						
*Eulemur coronatus*	2	C	40.05	59.00	125.0	-
*Eulemur fulvus*	3	C	44.32	71.90	120.0	159.0
*Eulemur macaco*	2	C	44.56	62.00	129.0	135.0
*Eulemur mongoz*	2	C	43.97	58.50	129.0	152.0
*Eulemur rubriventer*	1	C	48.50	85.50	123.0	126.0
*Hapalemur griseus*	2	D	39.17	49.60	142.5	132.0
*Varecia variegata*	3	D	47.30	88.00	132.5	108.3
*Lemur catta*	3	D	41.60	79.40	135.0	142.0
**Indriidae**						
*Propithecus verreauxi*	2	D	46.40	103.20	140.0	181.5
**Cheirogaleidae**						
*Microcebus murinus*	1	N	18.02	5.80	60.0	40.0
*Mirza coquereli*	1	N	27.02	15.75	87.0	86.0
*Cheirogaleus medius*	5	N	23.25	12.00	62.0	61.0
**Lorisiformes Galagidae**						
*Otolemur crassicaudatus*	2	N	37.95	44.60	135.0	135.0
*Otolemur garnettii*	3	N	35.51	49.00	132.0	140.0
*Galago moholi*	2	N	24.89	13.40	123.0	92.0
*Galagoides demidoff*	1	N	21.05	8.95	110.0	45.0
**Lorisidae**						
*Nycticebus pygmaeus*	2	N	24.68	20.00	188.0	133.0
**HAPLORHINI**						
*Tarsius syrichta*	1^3^	N	29.50	26.20	180.0	82.0

C, cathemeral; D, diurnal; N, nocturnal; CL, average cranial length (prosthion-inion) measured from this sample.

1 activity pattern according to Kirk, 2006 [2].

2 neonatal mass, gestation length, and weaning age obtained from [50], supplemented by data from other sources [51]–[55].

3 This is a 0-day-old *T. syrichta*. Two additional *T. syrichta* (one fetal and one 6-day-old) were studied for comparison to this 0-day-old infant. However, they were excluded from statistical analyses due to prematurity or, in the case of the 6-day-old, because the eyes had been removed prior to acquisition.

Because of the rarity of prosimian samples, especially *T. syrichta* and lorisids, four subadult samples were studied for descriptive purposes or metric comparisons, although they were excluded from calculation of regression models. These included two prenatal *Loris tardigradus* (47 mm CRL fetus; 39.6 mm CRL fetus,127 days gestation (full gestation: 167 days; gestation age calculated based on the date based on observed breeding behavior - S Zehr, personal communication)) and two additional *T. syrichta* (48.7 mm CRL fetus; 6-day-old infant). Two of the *Tarsius syrichta* (0 days postnatal; 6-day-old postnatal) and the 127 day-old fetal *L. tardigradus* were studied using micro-computed tomography (CT) with a Scanco vivaCT 75 scanner (55 kVp, 20.5 µm reconstructed voxel size) at Northeastern Ohio Medical University (NEOMED). The CT-scanned specimens were reconstructed using Amira 5.3 software (Visage Imaging, GmbH).

### Measurements

**Figure 1:**
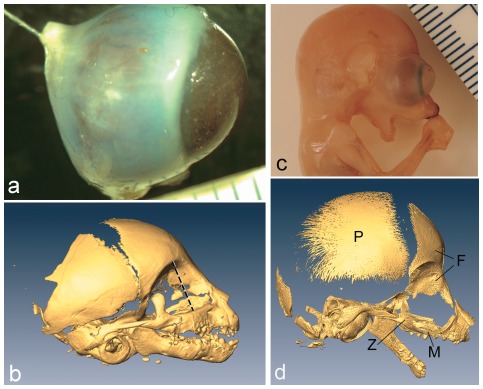
Eye measurements were taken by inserting a small gauge needle through the stalk of the optic nerve, as shown in *O. crassicaudatus* (1a). Using a syringe, the eyes were then injected with 10% buffered formalin until the external wall was smoothed of all wrinkles and the eye resisted further volumetric expansion. 1b) microCT reconstruction of perinatal *Tarsius syrichta*, showing the orbital aperture diameter measurement (dashed line), which was the maximum diameter in the parasagittal plane. c, d) Fetal slender loris (*L. tardigradus*), showing large extraorbital portion of the eye (c), as inferred by comparison to micro CT reconstruction of the same specimen (d). The frontal (F) and bone has an expanded orbital surface. M, maxilla; P, parietal; Z, zygoma. Ruler for 1a and 1c is in mm.

Osteological and soft tissue measurements were collected for this study. First, the eyes of the cadaveric sample were removed. All tissues surrounding the orbital rim were dissected away to provide an unobstructed view of the orbit and the anterior part of the zygomatic arch. Next, the skin over the posterior aspect of the skull was dissected open. Eyes were removed using microdissection tools and were cleaned of periorbital connective tissue. Extraocular muscles were removed or reflected. Eyes were visually inspected for damage. In some specimens, the sclera was damaged (small puncture hole) during removal. In these instances, the surface of the eye surrounding the puncture was dried with a paper towel and the hole was sealed using a fast-drying cyanoacrylate glue (“Hot Stuff” Special “T”). Most eyes were partially collapsed due to loss of internal fluids post mortem. In all such cases, the eyes were refilled with 10% formalin using a 10 cc syringe with a small gauge needle, as described in Kirk [2]. With the needle inserted and the eye maintained at full internal pressure (Figure 1a) measurements of the eye were taken using digital calipers to the nearest 0.01 mm. These included axial eye diameter (from anterior cornea to root of optic nerve), maximum transverse (equatorial) eye diameter, and minimum transverse eye diameter. For all analyses of transverse eye diameter, the average of the maximum and minimum values was used. All orbital and eye measurements were made twice; if the second measurement differed from the first by more than 10%, the measurement was repeated a third time, and the outlier was excluded. The average of the two measurements was then used for each specimen. All adult values for eye and orbit diameter were taken from Kirk [2], except for one adult *Otolemur garnettii*.

Using digital calipers, the following osteological measurements were made: cranial length (prosthion to inion) and orbital aperture diameter (diameter of the orbital aperture in a parasagittal plane – Fig. 1b). “Orbital aperture” is here defined, as in Kirk [2], as the margin of the bony orbit, that is, the crests of the lacrimal, maxillary, zygomatic and frontal bones that form the boundary between the orbital fossa and external facial skeleton.

**Figure 2:**
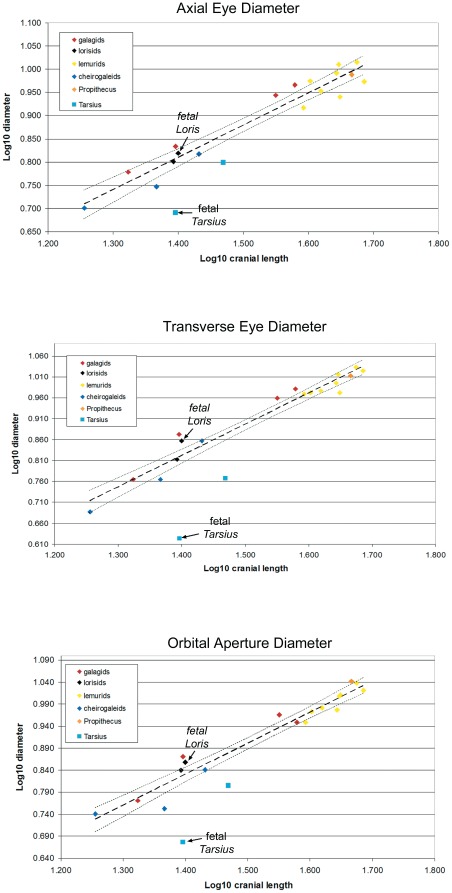
Relationship of Log10 eye and orbital diameters to Log10 cranial length in prosimians. The regression line is calculated from the strepsirrhines only. Thin lines indicate 95% confidence interval. Note that *Tarsius* is an outlier in each case.

The fetal and 6-day-old *T. syrichta* and the two fetal *L. tardigradus* were not included in the statistical analyses (see below). However, the fetal *T. syrichta* and the larger of the two fetal *L. tardigradus* were measured for a graphical comparison to neonates of other species (Figure 2).The 6-day-old *T. syrichta* was used to take measurements of the orbit for comparison to the younger tarsiers. Measurements of the 6-day-old *T. syrichta* were taken from cranial reconstructions using Amira software. This was necessary because the specimen was subsequently histologically processed for an different study (Smith, unpublished).

### Data analysis

Data were averaged for each of the eighteen species under study. A life history profile was created for each species, including neonatal body mass (grams), female adult body mass (grams), gestation length (days) and age at weaning (days). All life history variables were collected from the literature (Table 1). *Log_10_* transformations were made of all variables to obtain normality.

Relative size can be assessed using a variety of methods, such as ratios, regression residuals, or the geometric mean [26]. We employ two methods for comparing growth. First, the ratios of neonatal versus adult diameter were calculated for axial eye diameter, transverse eye diameter and orbital aperture diameter (Table 2). Neonate/adult ratios were also calculated for cranial length and body mass (Table 2). This method allows use of species-specific values to compare the proportional size of the eye to that of other somatic measurements.

**Table 2:** Eye measurements and ratios of the species studied.

Species	Neonatal AD (mm) +/− SD	Neonatal TD (mm) +/− SD	Neonatal OA (mm) +/− SD	Adult AD (mm)	Adult TD (mm)	Adult OA (mm)	ratio neonate/adult AD^1^	ratio neonate/adult TD^1^	ratio neonate/adult OA^1^	ratio neon./adult CL^1^	ratio neon./adult body mass^2^	ratio neon./adult brain mass^3^
*Eulemur coronatus*	9.43	9.38	9.40	16.1	16.1	18.4	0.59	0.58	0.51	0.48	0.05	-
*Eulemur fulvus*	10.03+/−0.42	10.38+/−0.14	10.27+/−0.18	16.7	17.6	19.7	0.60	0.59	0.52	0.48	0.03	0.42
*Eulemur macaco*	8.72	9.38	10.28	15.3	17.4	19.9	0.57	0.54	0.52	0.48	0.03	-
*Eulemur mongoz*	9.81	9.89	9.48	15.9	16.4	19.3	0.62	0.60	0.49	0.53	0.04	-
*Eulemur rubriventer*	9.41	10.59	10.50	18.2	18.1	20.4	0.52	0.59	0.51	0.56	0.04	-
*Hapalemur griseus*	8.27	9.33	8.88	13.8	14.1	15.4	0.60	0.66	0.58	0.58	0.07	-
*Varecia variegata*	10.36+/−0.57	10.79+/−0.47	10.9+/−0.37	18.5	19.4	22.2	0.56	0.56	0.49	0.45	0.03	0.31
*Lemur catta*	8.98+/−0.38	9.49+/−0.09	9.58+/−0.33	15.6	16.2	17.8	0.58	0.59	0.54	0.48	0.04	0.34
*Propithecus verreauxi*	9.73	10.30	11.00	17.7	17.8	19.5	0.55	0.58	0.56	0.55	0.03	-
*Otolemur crassicaudatus*	9.25	9.57	8.89	16.3	17.4	20.1	0.57	0.55	0.44	0.49	0.04	0.34
*Otolemur garnettii*	8.75+/−0.24	9.03+/−0.14	9.14+/−0.19	16.2	15.4	17.3	0.54	0.59	0.53	-	0.07	-
*Galago moholi*	6.82	7.46	7.42	13.4	13.8	15.0	0.51	0.54	0.49	0.58	0.07	-
*Galagoides demidoff*	6.00	5.82	5.90	10.1	9.6	11.6	0.59	0.61	0.51	0.58	0.15	0.44
*Microcebus murinus*	5.02	4.87	5.51	9.2	9.4	10.4	0.55	0.52	0.53	0.54	0.09	-
*Mirza coquereli*	6.56	7.19	6.94	13.0	13.0	15.0	0.50	0.55	0.46	0.53	0.05	-
*Cheirogaleus medius*	5.59+/−0.51	5.82+/−0.12	5.66+/−0.28	10.3	10.5	12.9	0.54	0.55	0.44	0.54	0.08	-
*Nycticebus pygmaeus*	6.32	6.5	6.92	15.5	14.9	17.1	0.41	0.44	0.40	0.47	0.07	-
*Tarsius syrichta*	6.30	5.86	6.38	17.3	17.9	18.6	0.36	0.33	0.34	0.74	0.22	-

AD, axial eye diameter; CL, cranial length (prosthion-inion); OA, orbital aperture diameter; TD, transverse eye diameter; SD, standard deviation (for species in which three or more specimens were measured).

1 Ratios calculated using infant data from the present study and adult data from [2];

2 Ratios calculated using data from Kappeler and Pereira [50];

3 Ratios calculated using data from [11].

Second, for each of three variables (eye axial diameter, eye transverse diameter, and orbital aperture diameter), the adult value (dependent variable) was compared with the neonatal value (independent variable) using least-squares regression. The residuals from these regression lines (hereafter referred to as “growth residuals”) for each species are provided in Table 3. Harvey et al. [11] used residuals from an ontogenetic dataset of brain masses in a similar manner. Positive values indicate a relatively greater-than-expected extent of postnatal growth for the variable in question. Conversely, negative growth residuals are indicative of a greater-than-expected extent of prenatal growth [11].

**Table 3:** Relative eye and orbital diameter and eye and orbit growth residuals in strepsirrhines.

Species	Relative axial eye diameter	Relative orbital aperture diameter	Relative transverse eye diameter	AD Growth Residual^1^	OA Growth Residual	TD Growth Residual
*Eulemur coronatus*	0.02457	−0.00076	−0.00087	−0.01494	−0.00139	−0.00204
*Eulemur fulvus*	0.02096	0.00658	0.01028	−0.02117	−0.00391	0.00012
*Eulemur macaco*	−0.04144	0.00534	−0.03546	−0.00901	0.00012	−0.02417
*Eulemur mongoz*	0.01371	−0.02575	−0.00815	−0.03453	0.01627	−0.01311
*Eulemur rubriventer*	−0.03379	−0.01147	−0.01023	0.03907	0.00320	0.00506
*Hapalemur griseus*	−0.02577	−0.01866	0.00401	−0.03483	−0.05801	−0.05772
*Varecia variegata*	0.01550	0.01246	0.00602	0.01168	0.02634	0.02845
*Lemur catta*	−0.00806	−0.00418	−0.00812	−0.01111	−0.02268	−0.00356
*Propithecus verreauxi*	−0.00599	0.02232	−0.00794	0.01498	−0.03330	0.00782
*Otolemur crassicaudatus*	0.03236	−0.00846	0.02529	−0.00267	0.05726	0.02445
*Otolemur garnettii*	0.02816	0.02399	0.02161	0.01539	−0.01898	−0.00679
*Galago moholi*	0.02655	0.04272	0.05384	0.02152	−0.00431	0.013578
*Galagoides demidoff*	0.02119	−0.00557	0.00033	−0.05534	−0.03250	−0.05453
*Microcebus murinus*	−0.00963	0.01244	−0.02669	−0.03193	−0.05507	0.00057
*Mirza coquereli*	−0.01497	−0.01171	0.01121	0.02229	0.02014	0.00093
*Cheirogaleus medius*	−0.03937	−0.05412	−0.03189	−0.02144	0.02873	−0.01561
*Nycticebus pygmaeus*	−0.00397	0.01484	−0.00324	0.11204	0.07809	0.09655

AD, axial eye diameter; OA, orbital aperture diameter; TD, transverse eye diameter. “Growth residuals” were calculated using least-squares regressions. For each variable, the adult value was regressed against the neonatal value in order to calculate an “expected” adult value. Residuals were calculated from these equations. Since the predicted y value estimates the adult eye size for a given eye size at birth, the residuals approximate how much growth occurs postnatally as opposed to prenatally (positive values indicate more postnatal growth; negative growth residuals indicate more prenatal growth.

In our analyses, we used the cranial length of each specimen as a surrogate for body size because body mass for the cadaveric samples was not always available. Cranial length is highly correlated with neonatal body mass derived from the literature (Table 4) and the two variables scale isometrically (*log10* prosthion-inion length = 0.99+0.349×*log10* neonatal body mass). Furthermore, all of the linear morphometric and life history variables considered in this analysis are significantly correlated with cranial length (Table 4). To correct for these independent correlations with cranial length, residuals were calculated using least-squares regressions of a series of dependent variables (including axial eye diameter, transverse eye diameter, orbital aperture diameter, gestation length, and weaning age) on cranial length (independent variable). Henceforth, these residuals are referred to as the “relative” value for the dependent variable in question. To provide a size-adjusted metric of neonatal body size, we also calculated “relative neonatal body mass” for each species as the residual from a least-squares regression of neonatal body mass (dependent variable) on adult female body mass (independent variable). Relative eye and orbit measures among strepsirrhines were compared to life history traits using multiple linear regression (Table 5). Significance was set at p</ = 0.05. Data analysis was carried out using SPSS version 15.0.

**Table 4:** Pearson Correlation Coefficients of the Log10 Cranial Length versus Log10 Life History Variables and Log10 Eye/orbit Measurements.

Comparison (all variables Log10 transformed prior to analysis)	Pearson Coefficients
PrIn : weaning	0.845; p<0.001
PrIn : gestation	0.573; p<0.02
PrIn : neon. mass	0.985; p<0.001
PrIn : AD	0.968; p<0.001
PrIn : TD	0.979; p<0.001
PrIn : OA	0.976; p<0.001

AD, axial eye diameter; OA, orbital aperture diameter; TD, transverse eye diameter.

**Table 5:** Multiple Regression of Eye and Orbit Diameter Vs. Life History Variables.

Relative^1^ AD (R^2^ = 0.15, p = 0.555)				
	Sign.	Zero-order correlation	Partial correlation	PGLS
RelNeoMass	0.784	0.085	−0.081	F = 0.31; p = 0.74
RelGestAge	0.246	0.382	0.332	F = 0.83; p = 0.46
RelWeanAge	0.805	0.210	−0.073	F = 0.07; p = 0.92

Relative TD (R2 = 0.21, p = 0.405)				
	Sign.	Zero-order correlation	Partial correlation	PGLS
RelNeoMass	0.863	0.180	0.051	F = 0.09; p = 0.91
RelGestAge	0.403	0.442	0.243	F = 1.97; p = 0.18
RelWeanAge	0.669	0.367	0.126	F = 1.34; p = 0.29

Relative OAD (R^2^ = 0.27, p = 0.271)				
	Sign.	Zero-order correlation	Partial correlation	PGLS
RelNeoMass	0.399	−0.025	−0.245	F = 0.01; p = 0.99
RelGestAge	0.126	0.472	0.429	F = 2.93; p = 0.09
RelWeanAge	0.887	0.320	−0.042	F = 1.64; p = 0.22

With *Nycticebus* excluded				
Relative AD (R^2^ = 0.37, p = 0.154)				
	Sign.	Zero-order correlation	Partial correlation	PGLS
RelNeoMass	0.357	0.085	−0.279	F = 0.82; p<0.461
RelGestAge	**0.050**	0.553	0.553	F = 5.65; p<0.01
RelWeanAge	0.807	0.075	0.075	F = 0.36; p = 0.261

Relative TD (R2 = 0.52, p = 0.037)				
	Sign.	Zero-order correlation	Partial correlation	PGLS
RelNeoMass	0.476	0.180	−0.217	F = 0.014; p<0.96
RelGestAge	**0.035**	0.649	0.587	F = 8.02; p<0.005
RelWeanAge	0.236	0.507	0.353	F = 3.21; p = 0.07

Relative OAD (R^2^ = 0.36, p = 0.167)				
	Sign.	Zero-order correlation	Partial correlation	PGLS
RelNeoMass	0.211	−0.023	−0.372	F = 2.27; p<0.14
RelGestAge	0.052	0.495	0.549	F = 3.49; p<0.05
RelWeanAge	0.826	0.277	0.068	F = 1.49; p = 0.26

1 Relative values are calculated as residuals from cranial length (AD, TD, OAD, gestation, weaning) or maternal body width (RelNeoMass).

In regression analyses, *T. syrichta* was excluded for several reasons. First, this study focuses on strepsirrhines, and the haplorhine *T. syrichta* is included for comparative purposes only. Second, tarsier eye and orbit scaling relationship differ substantially from the strepsirrhine sample (Fig. 2), and may obscure the estimate of strepsirrhine scaling relationships of the eye and orbit. One strepsirrhine, *Nycticebus pygmaeus*, falls outside the sample distribution in both weaning age and gestation length. It is the sole strepsirrhine species that is two standard deviations away from a linear regression line between gestation or weaning age and cranial length. Moreover, if *N. pygmaeus* is excluded, correlation coefficients with cranial length rise from 0.845 to 0.91 for weaning age and from 0.573 to 0.78 for gestation length. Therefore, we ran two regression analyses: one with *N. pygmaeus* included and with *N. pygmaeus* excluded.

Because closely related species are more likely to share anatomical similarities than more distantly related species, phylogenetic information should be considered in morphological analyses [27]. All phylogenetic analyses were run using R with the following packages: GEIGER (http://cran.r-project.org/web/packages/geiger/index.html) and APE (http://cran.r-project.org/web/packages/ape/index.html). Significance was set at p</ = 0.05 for all analyses. For this analysis we used a dated consensus phylogeny obtained from 10kTrees version 3 (http://10ktrees.fas.harvard.edu/). To examine the effect phylogeny may have on our statistical models, we estimated the parameter lambda (λ), which scales the off-diagonal elements of the variance-covariance matrix (corresponding to internal branches of the phylogeny) and serves as a measure of phylogenetic signal [28]. Lambda usually falls between zero and one. Non-phylogenetic signals are not statistically different from zero, while values greater than zero (or values above zero that are statistically different from zero) indicate that the given tree topology and branch lengths may account for some of the variation in the trait under a Brownian motion model of evolution. We used GEIGER to generate the maximum likelihood estimates of lambda for each variable. A χ^2^ squared distribution was used to evaluate if the maximum likelihood estimate of lambda is significantly greater than 0 and not significantly different from 1. In addition to estimating the parameter of lambda for each variable, we used a phylogenetic generalized least squares (PGLS) model to examine the relationship between morphometric and ontogenetic variables while controlling for phylogenetic non-independence. A PGLS model allows for an examination of trait evolution that may depart from strict Brownian motion, thus improving the estimation of the trait correlation. Phylogenetic adjustments via PGLS were accomplished using APE.

The relationship between activity pattern and relative eye size was assessed using a one-way analysis of variance (ANOVA). Species were categorized as nocturnal, diurnal, or cathemeral according to Kirk [2]. Significance was assessed at p<0.05.

## Results

Both eye and orbital diameters are highly correlated with cranial length in strepsirrhines as a group (Table 4). In separate bivariate plots of axial eye diameter, transverse eye diameter, and orbital aperture diameter versus cranial length (Figure 2), a close relationship is seen between cranial length and eye or orbit diameters among strepsirrhines at birth (Figure 2 also includes a late fetal *Loris tardigradus* that falls close to the regression line). In contrast, the 0-day-old *T. syrichta* falls below the 95% confidence interval for the strepsirrhine regression lines. Measurements of the fetal *T. syrichta* are similarly well below the 95% confidence interval.

In all species, adult orbital aperture diameter is approximately 1–3 mm larger than adult eye diameters (Table 2). By contrast, neonatal specimens have eye diameters that more closely approximate the values for orbital aperture diameter. Furthermore, in a broad range of lemuroids (*E. coronatus*, *E. fulvus*, *E. mongoz*, *E. rubriventer*, *H. griseus*, *M. coquereli*) and lorisoid (*O. crassicaudatus*, *G. moholi*, *G. demidoff*) species, one or both neonatal eye diameters exceed the diameter of the orbital aperture (Table 2; see also Figs. 1c, d). In most species, ratios of neonatal∶adult eye diameter range from 0.50 to 0.62. Ratios of neonatal∶adult orbital aperture diameter are smaller for these same species, ranging from 0.44 to 0.58.

Because only one neonatal tarsier was available for dissection, we also measured a late fetal specimen. The eye and orbital diameters of the late fetal specimen shows a similar pattern to that observed in the neonatal specimen (Figure 2). However, the oldest (6-day-old) infant *T. syrichta* appears to have proportionally larger orbits. Although damage to the cranium indicates some distortion may be present, measurements of CT-reconstructions suggest orbital aperture diameter is 11.45 mm. When the orbital aperture diameters of all three *T syrichta* specimens are compared to published data on adults [2], the orbital aperture diameters show progressive enlargement from fetal (23% of adult diameter) to 0-day-old (34%) to 6-day-old (61%). Eyes had been removed prior to acquisition of the 6-day-old specimen. In 0-day specimens, eye diameters related to published adult data in a similar way as seen in the orbit (Table 2).

Among strepsirrhines, ratios of neonatal/adult diameters indicate that eye and orbit diameters are more than 50% of the values for adults in most species (Table 2). In strepsirrhines excluding *Nycticebus*, mean neonatal axial eye diameter is 56.2% of the adult value and mean neonatal transverse eye diameter is 57.5% of the adult value. By comparison, mean neonatal orbital aperture diameter and cranial length are only 50.8% and 52.3% of the adult values, respectively. *N. pygmaeus* and *T. syrichta* are the only two species included here that deviate substantially from this pattern. In *N. pygmaeus*, the neonatal measurements for eye diameter, orbital aperture diameter, and cranial length are all between 40% and 47% of the adult values. In *T. syrichta*, neonatal eye and orbit diameters are only 33 to 36% of the adult values, while cranial length is 74% of the adult value (Table 2). In all but one strepsirrhine (*M. murinus*), the neonate/adult ratios for orbital aperture diameter are less than those for transverse eye diameter.

**Figure 3:**
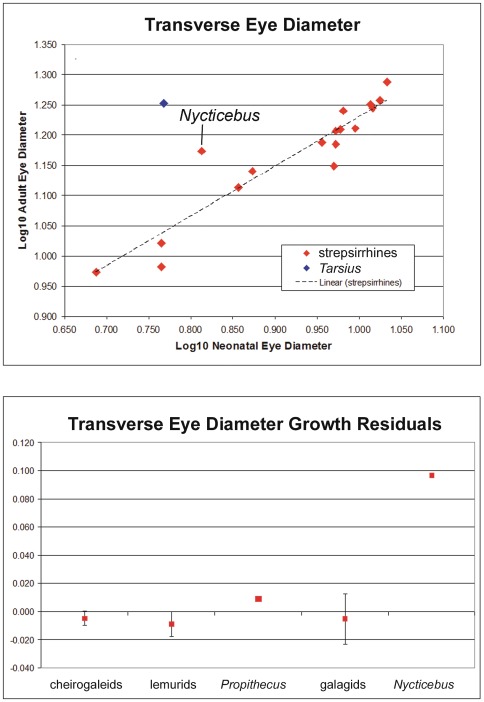
Regression of adult transverse eye diameter against neonatal transverse eye diameter (top). The bottom graph shows growth residuals for transverse eye diameter. The latter are the residuals (which are equivalent to the distance from the regression line), for strepsirrhines only, plotted in taxonomic groups.

Residuals derived from least-squares regressions of adult eye diameter on neonatal eye diameter provide results similar to those obtained with ratios (Figure 3, Table 3). In a bivariate plot of adult transverse eye diameter on neonatal transverse eye diameter (Figure 3), *N. pygmaeus* and *T. syrichta* lie well above most other species, indicating their eyes grow more postnatally than in other species. The residuals from this regression are also shown in Figure 3, calculated for the neonate strepsirrhine sample alone (Table 3). Growth residuals for cheirogaleids, lemurids and galagids are negative, while those for *P. verrauxi* and *N. pygmaeus* are positive (Figure 3). *N. pygmaeus* in particular departs strongly from all other species in this measure, while the growth residuals of *P. verreauxi* lie within the range of those for lemurids (Table 3).

**Figure 4:**
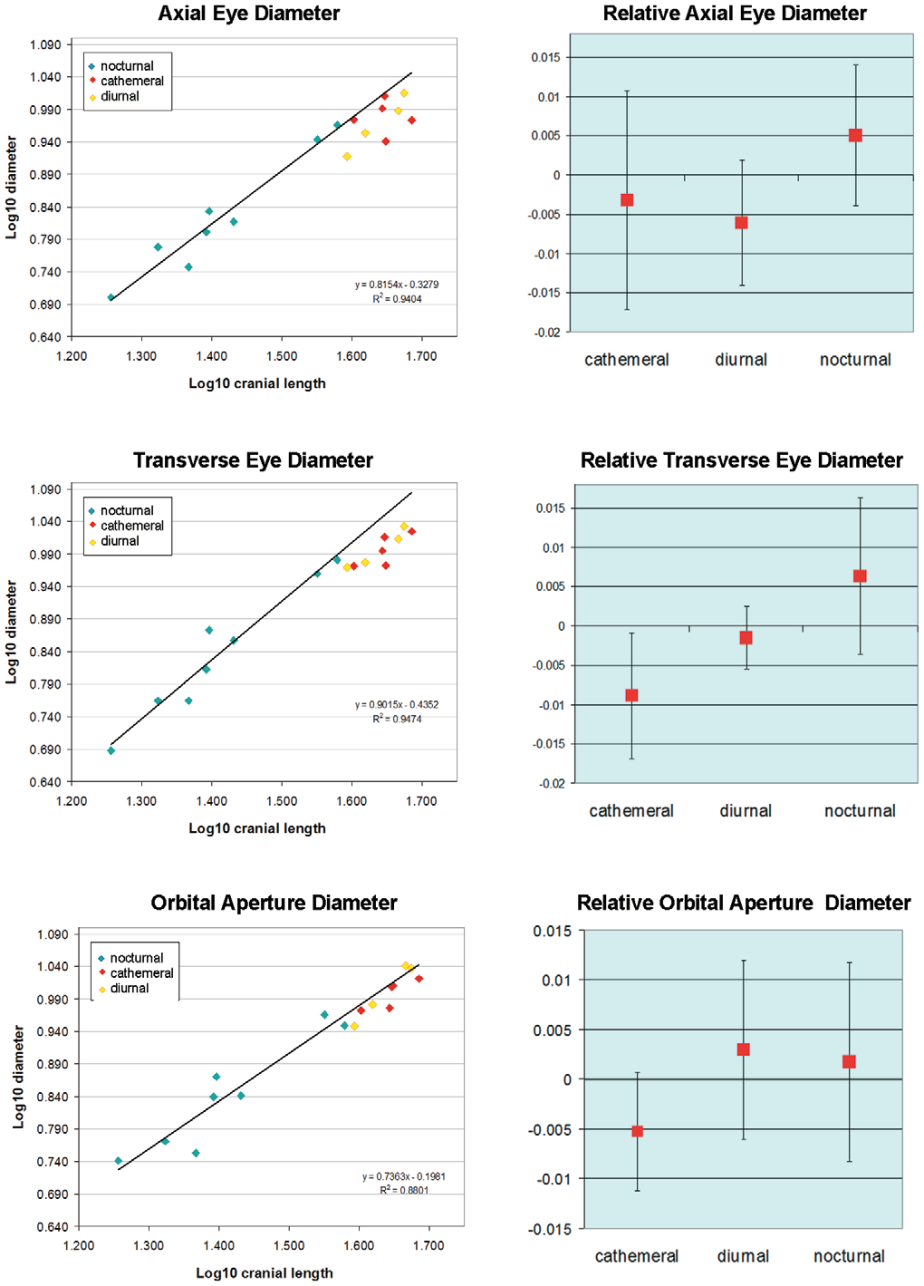
Eye diameter and activity pattern in newborn strepsirrhine primates. Left column, log10 transformed axial eye diameter, transverse eye diameter, and orbital aperture diameter plotted against cranial length in primates with different activity patterns. Note that all cathemeral and diurnal scale below the regression line for nocturnal primates. Right column: relative size (residuals) of the same measurements. Although no significant differences were found, nocturnal species show a trend toward relatively larger eye dimensions than cathemeral and diurnal species. The difference in orbital aperture dimensions is less apparent.

Results of ANOVA indicate there are no significant (p>0.05) morphometric differences among neonatal strepsirrhines according to activity pattern (axial eye diameter: F = 3.11; transverse eye diameter: F = 2.18; orbital aperture diameter: F = 1.15). However, plots of eye and orbit diameters against cranial length appear to reveal a scaling trend of higher eye and orbit diameter in nocturnal species compared to either cathemeral or diurnal species (Fig. 4). This is especially evident for eye diameters, for which all non-nocturnal species fall below a linear regression line for nocturnal species (Fig. 4). In relative size, nocturnal species likewise have a trend of having larger eye diameters. However, the residuals are highly variable in our sample (Fig. 4).

**Figure 5:**
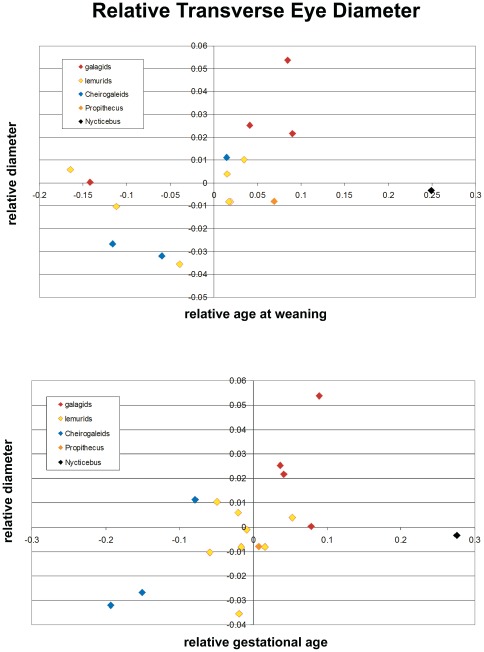
Relative transverse eye diameter (residuals calculated from regression of Log10 transverse eye diameter against Log10 cranial length) plotted against relative age at weaning (top) and relative gestational age (bottom). No relationship to relative neonatal body mass as apparent in our analysis (see Table 5).

Scatterplots indicate a positive association between relative transverse eye diameter and relative weaning and gestational age, with *Nycticebus* as a distinct outlier (Figure 5; and see above). Results of multiple linear regression indicate most of the variance in relative eye size is explained by relative gestation length, although correlations are not significant (Table 5). However, if the outlier (*Nycticebus*) is excluded, relative gestation age has a significant positive correlation with relative eye diameter in strepsirrhines. Based on the partial correlations, relative gestation length explains most of the variance in eye (55%, 59%) and orbit (55%) diameter (Table 5). Partial correlations of relative weaning age and relative neonatal mass to relative eye or orbit diameter are not significant (Table 5).

Results of the test to determine the influence of phylogeny on metric and life history variables revealed a strong influence of evolutionary history on some but not all variables. Relative gestation and weaning age seem to be under strong phylogenetic influence according to calculated λ values, while no such influence was detected for eye or orbit diameters (Table 6). Accordingly, all tests using life history data were repeated using phylogenetic corrections. When PGLS regressions are used to control for phylogenetic non-independence, the relationships between relative gestation length and (1) relative transverse eye and (2) orbital aperture diameter remain significantly correlated (Table 5).

**Table 6:** Lamba values for eye, orbit, and life history variables under study.

	AD	OA	TD	Weaning	Gestation Length
lambda [ML]	0.36	0.20	0.49	0.67	1
lower bound : 0.000	P>0.1	P>0.5	P>0.1	P>0.5	P<0.0001
upper bound : 1.000	P<0.001	P<0.0001	P<0.05	P<0.05	P = 1

## Discussion

### Age changes in eye and orbit diameter

Little is known regarding the timing of eye and orbit growth in primates. For most of the primates studied here, results suggest more than half of the total growth in eye diameter occurs prenatally, with *N. pygmaeus* and *T. syrichta* as exceptions to this trend. Eye dimensions have been studied in relatively few other mammals at the newborn age. Although previous works use a variety of techniques, a survey of neonate/adult ratios acquired from such studies can provide a comparative perspective. In some mammals, axial eye diameter grows more postnatally than prenatally (e.g., *Meriones* and *Oryctolagus* – Table 7). In this respect, gerbils and rabbits more closely resemble *N. pygmaeus*, in which neonates have eyes with axial lengths that are 41% of the adult value. In contrast, other non-primate mammals exhibit more prenatal than postnatal growth of the eye. Norrby [29] graphically presented growth data on axial eye diameter in rats (*Rattus norvegicus*) that range from about 4.5 mm at birth to about 7 mm at adulthood, indicating that ∼64% of axial eye diameter is established at birth. Data on the domestic dog (*Canis familiaris*) and the tree shrew (*Tupaia glis*) also suggest eye diameter may be more than half grown in infants, though in both cases, the initial measurements were taken weeks after birth [30], [31].

**Table 7:** Published ontogenetic data on axial eye diameter for mammals.

	AD (mm)			ratio	
Species	Diameter range (age range)	newborn	adult	neonatal/adult	references
*Canis domesticus*	12.7 to 19.5	-	19.5		[30]
	(2 to 52 weeks)	-	-		
*Meriones unguiculatus*	2.54 to 6.11*	2.54	6.11	0.42	[56]
	(P0 to P100)				
Oryctolagus cuniculus	7.01 to 16.27			0.43	[57]
	(P1 to P56)				
*Callithrix jacchus*	7 to 11	7	11	0.64	[33]
	(neonate to adult)				
*Macaca mulatta*	13.1 to 18.9	13.1	19.4**	0.68	[15]
	(P1 to 4 years)				[16]
*Homo sapiens*	Newborn to adult	16.5, 17.02	24.2	0.68/0.70	[17], [32]

* several diameters were measured from the gerbil eye. The description of “AP” length matches AD as measured in this study.

** , a longitudinal study [16] compared newborn AD to that of 4-year-old macaques, yielding a ratio of 0.69. If compared adult data from a different study [15], the ratio is 0.68.

Thus, published data suggest that eye growth patterns vary substantially among mammals. The most common pattern observed among strepsirrhines is for most growth in eye diameter to occur prenatally. Anthropoid primates may have an even greater extent of axial eye diameter established at birth (and thus more prenatal eye growth) compared to prosimians, although few species have been studied to date. Data published for *Homo* [32], *Macaca* [16] and *Callithrix* [33] have neonatal/adult axial eye diameter ratios (0.64–0.70; Table 7) that exceed the range for our prosimian sample (0.36–0.62; Table 2).

The two species with comparatively undersized eyes at birth, *N. pygmaeus* and *T. syrichta*, require special consideration here. Despite the relatively small diameters of the eye and orbit in the fetal and newborn *T. syrichta*, there is indirect evidence that the *pace* of orbit growth may be increasing in late fetal and early postnatal periods. First, the proportionally large orbit of the 6-day-old *T. syrichta* compared to the fetal and 0-day-old specimens suggests progressive early postnatal eye enlargement. Second, a rapid pace of late fetal eye growth is also indicated by published volumetric data on *T. bancanus* [8]. Whether the early postnatal eye/orbit growth rate is similarly rapid in *N. pygmaeus* cannot be assessed at present. However, an explanation for the more limited growth in diameter compared to other primates is required. Both *T. syrichta* and *N. pygmaeus* are distinguished as two of the primate species with the relatively largest orbits as adults [2]. In this light, relative eye size might be subject to selection based on its contribution to overall head size. Even the smallest-bodied primates have dimorphism relating to obstetric demands [34]; head size is surely an important selection factor since its growth outpaces that of the overall body mass (Table 2). When considering that postnatal growth of the eye in tarsiers is suggested to actually exceed that of the brain (see further discussion in [35], [36]), the possibility is raised that, perhaps for both lorisids and tarsiers, *eye* size may be constrained in accordance with spatial limits imposed by parturition.

### Implications for growth of the orbit, eyes, and face

**Figure 6:**
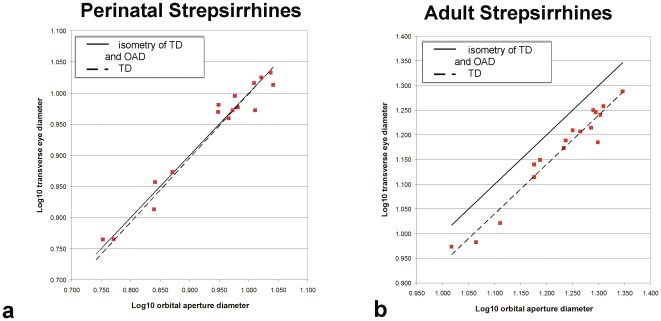
Regression of transverse eye diameter to orbital aperture diameter in neonatal (6a) and adult (6b) strepsirrhines. The solid regression line in each graph is hypothetical, showing the relationship if transverse diameter (TD) is equal to orbital aperture diameter (OAD). Note regression line slopes of TD to OAD (dashed lines) at both ages are similar to the hypothetical (solid) line. However, the TD of adults is smaller than OAD across species. Neonatal data from this study. Adult data are from this study (*O. garnettii*) and a previous report [2].

In adult strepsirrhines, orbital aperture diameter is greater than transverse eye diameter, but the diameters scale similarly to each other (Fig. 6; [2]). By comparison, orbital aperture diameter is nearly identical to transverse eye diameter in the neonatal specimens examined here (Fig. 6a). Thus, the eye is rather snugly nested within the orbit at birth, and even expanded beyond its limits (and see [4]). In the fetal *L. tardigradus*, (Figs. 1c,d) half or more of the eye protrudes from the orbit. This indicates that eye growth can outpace orbital expansion and ossification in utero, suggesting a disassociation of eye and orbit growth rates. However, subsequently eye and orbital growth rates diverge. In most strepsirrhines, growth of transverse eye diameter is ∼58% complete at birth while growth of the orbital aperture is only ∼51% complete (see ratios, Table 2). Accordingly, greater postnatal growth of the orbital aperture compared to the eye ultimately leads to the adult strepsirrhine pattern, in which the diameter of the orbital aperture always exceeds the transverse diameter of the eye (Fig. 6; [2]). Based on apparently closer association between eye size and orbit diameters at birth (Fig. 6a), growth of the eye itself may drive early (fetal and early postnatal) increase in orbit size (and may be important to orbital orientation [8]). Subsequently, eye diameters clearly increase at a different rate compared to that of the orbit (Fig. 6b).

The closer relationship of soft tissue (eye) and osseous (orbital aperture) diameters at birth compared to adult ages is consistent with growth patterns described broadly for vertebrates. Specifically, early expansion of the orbit follows eye growth; later in development eye growth slows while orbital growth continues [37]. Experimentally increasing [38] or decreasing [39] eye size in vertebrates affects orbit size. However, the orbit is independent, especially across postnatal development, in that it continues to grow even in the absence of the eye [40], [41]. Thus, the eye and orbit are argued to have different “growth potentials” [37].

### Neonatal eye size, activity pattern and life history

Primate orbit size is known to relate to activity pattern: nocturnal species have relatively larger orbital aperture diameters than cathemeral or diurnal species as adults [2]. Orbit diameter is more divergent among these groups than diameter of the eye itself [2]. Here, we show that the diameters of the orbital aperture and eye do not differ significantly among perinatal strepsirrhines according to activity pattern. However, the trends of the relationship of eye and orbit diameter to cranial size resemble those shown for adult strepsirrhines by Kirk [2]. This result suggests that differences in form between nocturnal and other strepsirrhines may be beginning to appear perinatally, with a more pronounced divergence in morphology manifested postnatally.

While the influence of ecological factors such as activity pattern may be subtle at birth, other factors may strongly influence eye and orbit development prenatally. One such factor is somatic growth. Eye and orbit diameters are highly correlated with absolute cranial length: larger heads house larger eyes. Our head size measurement spans both facial and neurocranial regions, making possible an association of eye and orbit diameters with either or both component. Data on brain weights could resolve the primary correlation, but these are available for only some of the species under study (Table 2). Based on existing data, it is clear that the association among eye and many somatic variables is inconsistent. For example, species in which the eye diameter is greatest at birth (∼60% or more of adult diameter) represent strepsirrhines that vary in body weight, including those with the relatively largest neonates (*Galagoides*) as well as the relatively smallest (*Eulemur* spp)(Table 2). *T. syrichta* provides another striking example. In body weight [42], muscular weight [20], and the extent of cranial length achieved at birth (74%, Table 2), tarsiers are precocial. However, transverse and axial eye diameters of the late fetal and neonatal tarsier appear to be relatively undersized compared to other taxa. The lack of a consistent association of eye growth with somatic growth is in keeping with other cranial structures that seem to disassociate from other body regions. In other words, the manner in which structures are interrelated changes during development, a concept called *modularity* [43]. Our results suggest the well-documented modularity of growth among primates (for example, of brains, teeth, and body mass [22], [43], [44]), also pertains to the growth of eye diameter.

While no correlation exists between relative neonatal weight and eye or orbit diameters, eye and orbit diameters have positive correlations with some, but not all, of the life history traits under study. Mass of neonates relative to that of adult females is commonly used to estimate the amount of maternal investment during pregnancy [45], [46]. Relative neonatal mass, which may be taken to indicate investment in relatively large (or small) newborn body mass, has no detectable influence on eye or orbit diameters. Instead, eye and orbit diameters correlate with a life history variable relating to the *pace* of development. For example, as relative gestation age increases, so does the relative diameter of the eye and orbit at birth. Some deviations from this pattern might be expected among lorisids, which have a notably long gestation length, slowly developing brains (low neonatal brain mass relative to adult brain mass [11]), as well as other heterochronic differences compared to most strepsirrhines [47], [48]. The pygmy slow loris falls out of the distribution of other strepsirrhines when relative eye size is related to relative gestation length or relative weaning age (Figure 4). When *N. pygamaeus* is removed from the analysis, correlations are significant, even after phylogenetic correction (Table 5). The correlation of eye and orbit size to weaning age is also positive. This follows the pattern reported for brain mass in anthropoids: species in which brain growth occurs at an earlier age tend to wean late [22]. However, the correlation between relative eye size and relative weaning age is not significant. Analysis of a broader taxonomic sample of primates may be needed to establish whether a significant correlation exists between eye diameter and weaning age.

### Conclusions

Compared to many other mammals, primates tend to invest more in prenatal growth for their offspring [11], [19]. The developmental strategy of relatively long gestations is arguably a great benefit to neural development. It is regarded as one explanation for the relatively large neonatal brain size of precocial mammals [19], [49]. Our findings suggest that parallels exist between primate eye and brain growth in that the much growth of eyes (like brains) occurs prenatally. Moreover, the strepsirrhine species with the most undersized eye and orbit diameters at birth (lorisids) also defer more brain growth to the postnatal period [11]. Thus, the close relationship of eye diameter and gestation length may indicate that eyes follow a neural growth trajectory, even in the case of exceptions. Results of our analyses appear to reveal a relationship between the nature of maternal investment and eye development. Specifically, time may be invested in feeding the young during gestation or postnatally (until weaning). Results here indicate eye size relates more strongly to the duration of gestation than to postnatal care provided until weaning.

If life history traits such as prolonged gestation influence eye growth, by extension it influences the ontogeny of facial form. Across all species, eye growth is likely to exert an early influence on facial morphogenesis. Growth of the eye precedes orbital growth, and this timing affects facial form early in development (i.e., prenatal and early postnatal stages) [8]. Our results also support the contention that eye and orbit growth, though highly correlated, have some autonomy [37]. Some of the most salient patterns in the comparative morphology of the adult primate orbit, such as the enlarged orbits of nocturnal primates [2], [4], mainly emerge postnatally, when orbital expansion outpaces eye growth.
